# Double posteromedial portals versus single posteromedial portal for arthroscopic management of popliteal cysts

**DOI:** 10.1186/s13018-023-04132-6

**Published:** 2023-09-04

**Authors:** Rui Ma, Zheyue Zhu, Dan Liu, Kunzheng Wang, Pei Yang

**Affiliations:** Department of Bone and Joint Surgery, The Second Affiliated Hospital of Xi’an Jiaotong University, No. 157 Xiwu Road, Xi’an, 710004 Shaanxi People’s Republic of China

**Keywords:** Popliteal cyst, Arthroscopy, Posteromedial portal, Internal drainage

## Abstract

**Background:**

As a common disease in orthopedic clinics, popliteal cysts often coexist with intra-articular lesions. Compared with traditional open surgery, arthroscopic treatment of popliteal cysts is less traumatic, and intra-articular lesions can be treated. The ‘one-way valve’ mechanism of the popliteal cyst can be removed by expanding the communication between the articular cavity and the cyst to avoid cyst recurrence. In terms of arthroscopic techniques, the comparison of clinical effects between the double posteromedial portal (DPP) and single posteromedial portal (SPP) has rarely been studied. The purpose of this retrospective study was to compare the clinical effects of DPP and SPP.

**Methods:**

A total of 46 consecutive patients with symptomatic popliteal cysts who underwent arthroscopic treatment were included in this study and followed for approximately 1 year. All patients were divided into two groups according to the arthroscopic portals (DPP group and SPP group). The cyst size, Lysholm score and Rauschening–Lindgren (R–L) grade were evaluated before the operation for all patients, and the intra-articular lesions, operative time and complications were recorded after operation. At the last follow-up, the Lysholm score and R–L grade were recorded, and magnetic resonance imaging was used to evaluate the outcome of the cyst. The clinical data of the two groups was statistically compared and analyzed.

**Results:**

There were no significant differences in preoperative cyst size, Lysholm score or R–L grade between the two groups (*P* > 0.05). The operation time of the DPP group (67.52 ± 18.23 min) was longer than that of the SPP group (55.95 ± 16.40 min) (*P* = 0.030), but the recurrence rate of cysts in the DPP group (0%) was obviously lower than that in the SPP group (19.0%) (*P* = 0.046). There were no significant differences in the Lysholm score, R–L grade or complication rate between the two groups at the last follow-up (*P* > 0.05).

**Conclusion:**

Arthroscopic treatment of popliteal cysts using double posteromedial portals was a safe and effective surgical method.

*Trial registration*: ChiCTR, ChiCTR2200060115. Registered 19 May 2022, https://www.chictr.org.cn/showproj.html?proj=133199

## Background

Popliteal cysts are cystic masses behind the popliteal fossa of the knee joint and are very common in orthopedic clinics. They mainly cause pain in the posterior knee joint, local swelling and a sense of distention in the popliteal area. Under some circumstances, popliteal cysts affect knee flexion and extension to varying degrees, and rarely may cause compartment syndrome and gastrocnemius atrophy [1]. Large popliteal cyst can even compress the popliteal vessels and nerves, causing ischemia or thrombosis, and peripheral neuropathy [2]. Sometimes, rupture of the popliteal cyst can cause pain and swelling of the leg [3].

Symptomatic popliteal cysts are recommended for surgical treatment [4]. Traditional open surgery is usually associated with major trauma, slow recovery, a high risk of infection, and noticeable wound scars, which affects postoperative rehabilitation exercises. There are important nerves and blood vessels that pass through the popliteal fossa, so open surgery is risky [1]. In addition, open surgery only focuses on the cyst itself and does not consider the intra-articular pathology and unidirectional valve mechanism. Open surgery is associated with an increased risk of recurrence, and joint functional recovery is not ideal [5].

Popliteal cysts are often accompanied by intra-articular pathology [6], as they generate too much joint synovial fluid and increase the pressure of the joint cavity. Therefore, a ‘one-way valve’ flow mechanism forms [7], and the joint synovial fluid can enter the cyst cavity but cannot return to the articular cavity. Arthroscopic surgery can handle intra-articular pathology at the same time and eliminate the ‘one-way valve’ mechanism by enlarging the traffic port to reduce the risk of recurrence of the cyst [8]. Compared with traditional open surgery, arthroscopic surgery has the advantages of less trauma, fast recovery, patient tolerability, fewer postoperative concerns and low rates of infection and rigidity [9]. In addition, arthroscopic surgery is considered to have the advantages of a low postoperative recurrence rate, minimal postoperative pain, a good knee function score, less intraoperative bleeding, and a small surgical incision [10].

The arthroscopic portals for implementing arthroscopic surgery of popliteal cysts involve double posteromedial portals (DPP) and single posteromedial portal (SPP).However, whether DPP or SPP is optimal for satisfactory clinical effects is unclear. The aim of this study is to compare the clinical effects of two methods of arthroscopic portals for popliteal cysts (DPP and SPP) through a retrospective study to provide a theoretical guide for clinical surgery.

## Methods

### Patients

This was a retrospective case‒control study. From March 2019 to April 2022, a total of 54 patients were admitted to the hospital for treatment of popliteal cysts in the Department of Bone and Joint Surgery of our hospital. Patients who had unilateral initial symptomatic popliteal cysts and were treated with arthroscopic surgery were included in this study. The exclusion criteria were as follows: (1) history of joint surgery or open surgery for cysts; (2) ligament injuries; (3) rheumatoid arthritis, septic arthritis, gouty arthritis; (4) severe coagulation disorders; and (5) incomplete clinical data. One patient with a history of arthroscopic surgery, 1 with a history of open surgery, 2 with recurrent cysts, 2 with an anterior cruciate ligament (ACL) injury and 9 with incomplete clinical data were excluded. A total of 46 cases were selected and divided into two groups according to the arthroscopic portals used in the arthroscopic surgery: the DPP group (25 cases) and the SPP group (21 cases). All patients were followed for approximately 1 year. This study was approved by the medical ethics committee of the Second Affiliated Hospital of Xi’an Jiaotong University.

### Surgery

**Fig. 1:**
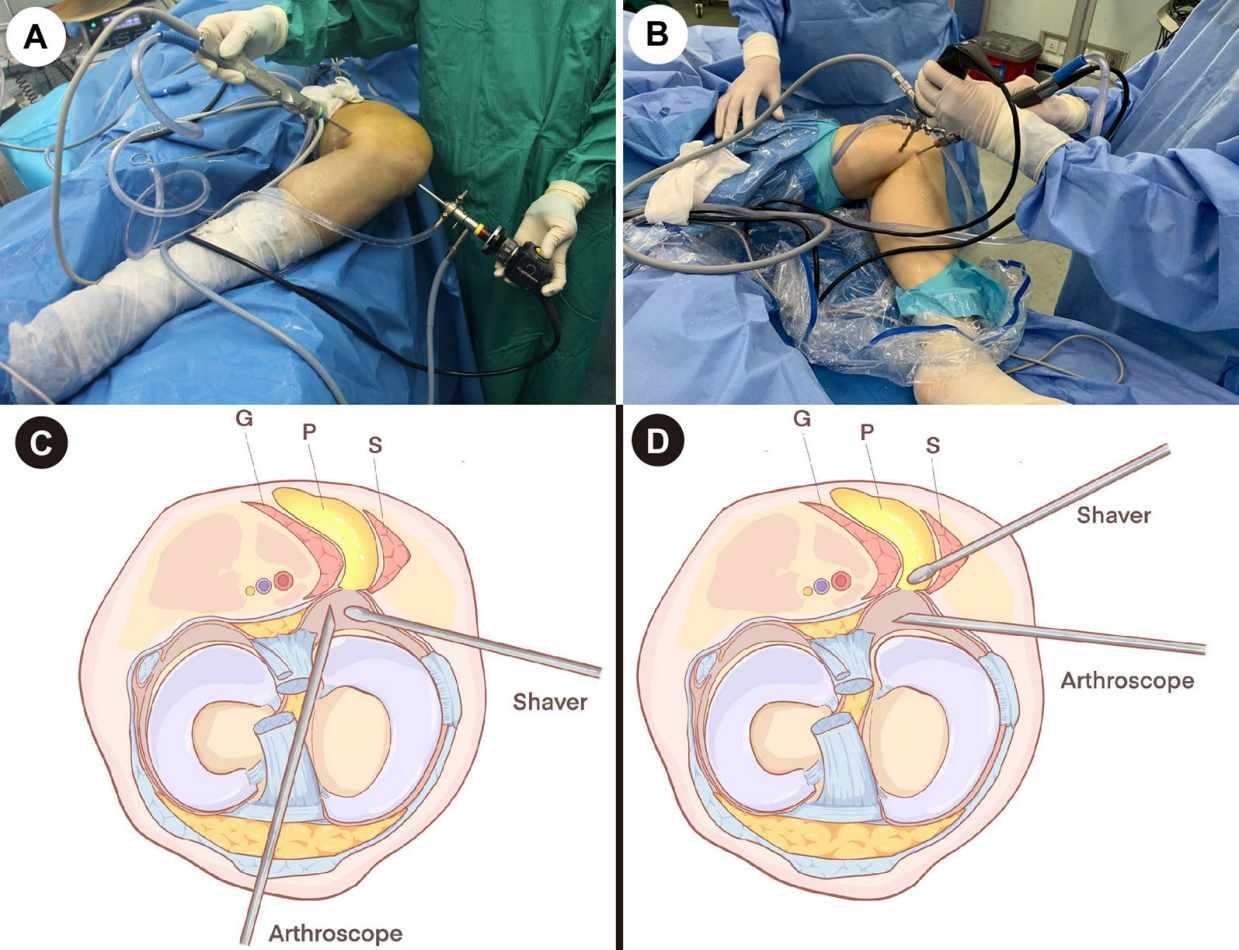
Photos and schematic diagrams of intraoperative positions and arthroscopic portals of the two groups (G, gastrocnemius; P, popliteal cyst; S, semimembranosus)

**Fig. 2:**
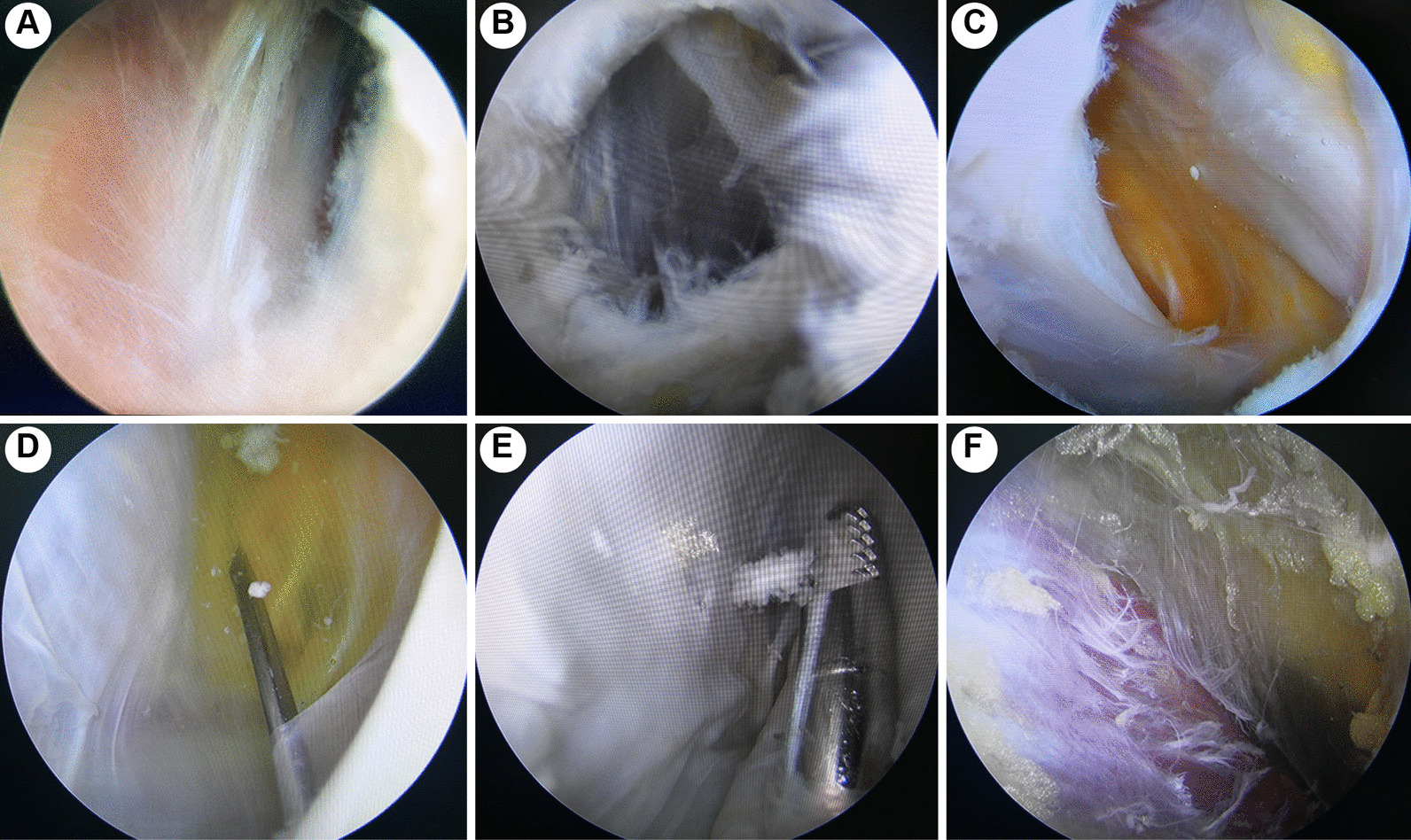
Arthroscopic images of popliteal cyst treatment by double posteromedial portals: **A** after removing the folding portion of the posterior joint capsule, the fissure of the gastrocnemius-semimembranosus bursa was exposed; **B** the communication port was expanded by the shaver through the posteromedial portal; **C** the cyst cavity was observed through the anterolateral portal; **D** a needle was placed from the outside to establish an auxiliary posteromedial portal; **E** the cyst cavity was observed by the posteromedial portal, and the cyst wall was removed through the auxiliary posteromedial portal; **F** the cyst was already removed, and the muscle and adipose tissues were exposed

The patients were placed in a supine position and given combined spinal-epidural anesthesia or general anesthesia. The arthroscopic optics used in the arthroscopic surgery was Smith and Nephew and the lens was 30°. The patients were banded with a pneumatic tourniquet (40 kPa) at the base of the thigh. Routine anterolateral and anteromedial portals were established to observe and treat intra-articular pathologies (degenerative cartilage damage, medial meniscal tear, lateral meniscal tear, synovitis and synovial hypertrophy and loose body). The knee was placed into a figure-of-four position (Fig. 1A, C), and a routine posteromedial portal was established from outside to inside. The fold portion of the articular capsule was shaved by the shaver to find the opening fissure of the gastrocnemius-semimembranosus bursa (Fig. 2A). The communication port was expanded by the shaver (Fig. 2B). The medial head of the gastrocnemius was located, and an exchange rod was placed. Then, the arthroscopic lens was placed into the posteromedial compartment and entered the cyst cavity along the medial head of the gastrocnemius (Fig. 2C). The cyst cavity was observed through the posteromedial portal, and an auxiliary posteromedial portal was established 3 cm distal to the posteromedial portal by a puncture needle from outside to inside (Fig. 2D). Under a figure-of-four position, the cyst wall was removed by the shaver using double posteromedial portals (Figs. 1B, D and 2E, F). The skin incisions were sutured. A drainage tube was placed through the posteromedial portal, and the lower limb was compressed by an elastic bandage for 14 days postoperatively.

### Postoperative treatment

All patients were given cefazolin sodium to prevent infection within 24 h, and were given celecoxib capsules to relieve pain within 2 weeks. All patients could get out of bed using crutches on the second day after the operation, and perform muscle strength training and joint motion training step by step.

### Clinical outcomes

All patients underwent magnetic resonance imaging (MRI) examination to measure the cyst diameter, Lysholm score and Rauschening–Lindgren (R–L) grade [11]. The operation time and the intra-articular pathologies were recorded. At the follow-up period, MRI examination was performed, and the Lysholm score and R–L grade were assessed. During the follow-up period, all complications (hematoma formation, fluid infiltration, poor wound healing, neurovascular injury, deep venous thrombosis, infection) were recorded.

### Statistical analysis

SPSS 20.0 statistical software was used for statistical analysis. Measurement data are presented as the mean ± standard deviation. Differences between groups were compared using two independent sample *t*-tests (measurement date) and Fisher's exact probability tests (categorical data). *P* < 0.05 was considered to be significantly different.

## Results

### Patient demographics and characteristics

A total of 46 patients who completed the follow-up were included in this study. Twenty-five patients were in the DPP group, and 21 patients were in the SPP group. There were no significant differences in age (*P* = 0.713), gender (*P* = 0.883), duration (*P* = 0.778) or size of the cyst (*P* = 0.512) between the two groups (Table 1).

**Table 1:** Patient demographics, characteristics and intraoperative data of the two groups

	DPP group (*n* = 25)	SPP group (*n* = 21)	*P* value
Age (years)	55.40 ± 8.61	54.43 ± 9.12	0.713
Gender (*n*.)			
Male	9	8	0.883
Female	16	13	
Duration (months)	12.42 ± 10.58	13.33 ± 11.23	0.778
Cyst size (cm)	11.44 ± 5.36	12.52 ± 5.76	0.512
Operation (min)	67.52 ± 18.23	55.95 ± 16.40	0.030
Associated articular pathologies			
Degenerative cartilage damage	14 (56.0%)	13 (61.9%)	0.876
Medial meniscal tear	8 (32.0%)	7 (33.3%)	
Lateral meniscal tear	5 (20.0%)	4 (19.0%)	
Medial and lateral meniscal tear	1 (4.0%)	0 (0.0%)	
Synovitis and synovial hypertrophy	4 (16.0%)	6 (28.6%)	
Loose body	2 (8.0%)	3 (14.3%)	

### Intraoperative data

The operation times of the DPP group and the SPP group were 67.52 ± 18.23 min and 55.95 ± 16.40 min, respectively. The operation time of the DPP group was obviously longer than that of the SPP group (*P* = 0.030). Intraoperative arthroscopy revealed found articular pathologies including degenerative cartilage damage (56.0% in the DPP group and 61.9% in the SPP group), medial meniscal tears (32.0% in the DPP group and 33.3% in the SPP group), lateral meniscal tears (20.0% in the DPP group and 19.0% in the SPP group), medial and lateral meniscal tears (4.0% in the DPP group and 0% in the SPP group), synovitis and synovial hypertrophy (16.0% in the DPP group and 28.6% in the SPP group) and loose bodies (8.0% in the DPP group and 14.3% in the SPP group). There was no significant difference in the frequency of associated articular pathologies between the two groups (*P* = 0.876) (Table 1).

### Knee functional scores

Two groups of patients were followed for approximately 1 year. There were no significant differences in the follow-up period (*P* = 0.128) between the DPP group (13.6 ± 1.8 months) and the SPP group (12.7 ± 2.0 months) (*P* = 0.128). Before the operation, no significant differences in the Lysholm score (*P* = 0.325) and R–L grade (*P* = 0.387) were found. The Lysholm score of the DPP group increased from 64.5 ± 9.2 points preoperatively to 83.4 ± 8.0 points postoperatively, and the Lysholm score of the SPP group increased from 62.0 ± 7.7 points preoperatively to 82.8 ± 7.8 points postoperatively, but the postoperative Lysholm scores between the DPP group and the SPP group showed no significant difference (*P* = 0.789). At the last follow-up, the R-L grade of the DPP group was as follows: I grade 9 cases, II grade 7 cases, III grade 9 cases; the R-L grade of the SPP group was as follows: I grade 4 cases, II grade 9 cases, III grade 8 cases. The R-L grade of the two groups showed no significant difference (*P* = 0.638).

### MRI outcome

From the results of the MRI analysis, no cyst recurrence was found in the DPP group, but 4 cyst recurrences were found in the SPP group (Table 2). In the cases of cyst shrinkage, the DPP group had 8 cases, and the SPP group had 8 cases. In the case of cyst disappearance, the DPP group had 17 cases, and the SPP group had 9 cases. The cyst recurrence rates of the DPP group and the SPP group rates were 0% and 19%, respectively, which showed a significant difference (*P* = 0.046) (Table 3).

**Table 2:** Knee functional scores of the two groups

	DPP group (*n* = 25)	SPP group (*n* = 21)	*P* value
Follow-up period (months)	13.6 ± 1.8	12.7 ± 2.0	0.128
Lysholm score			
Pre-operative	64.5 ± 9.2	62.0 ± 7.7	0.325
Post-operative	83.4 ± 8.0	82.8 ± 7.8	0.789
R–L grade (pre-operative)			
Grade 0	0	0	0.387
Grade I	9	4	
Grade II	7	9	
Grade III	9	8	
R–L grade (post-operative)			
Grade 0	10	7	0.638
Grade I	11	12	
Grade II	4	2	
Grade III	0	0

**Table 3:** MRI outcome and complications

	DPP group (*n* = 25)	SPP group (*n* = 21)	*P* value
MRI outcome			
Disappearance	17	9	0.046
Shrinkage	8	8	
Recurrence	0	4	
Complications (%)	2 (8.0%)	1 (4.8%)	0.658
Hematoma formation	0	0	
Fluid infiltration	2	1	
Poor wound healing	0	0	
Neurovascular injury	0	0	
Deep venous thrombosis	0	0	
Infection	0	0	

#### Complications

In addition to 2 cases of fluid infiltration in the DPP group and 1 case of fluid infiltration in the SPP group, no other complications occurred. The fluid infiltration improved after pressure bandaging and cold compression. The incidence rates of complications between the two groups were not obviously different (*P* = 0.658).

## Discussion

Popliteal cysts are often associated with intra-articular pathologies, such as meniscus injury, osteoarthritis, articular cartilage injury and loose body [12–15]. These lesions could serve as a primary source of pathological synovial fluid. A “one-way valve” flow mechanism formed, and continuous unidirectional flow between the posteromedial compartment and the gastrocnemius-semimembranosus bursa occurred, supporting the development and persistence of popliteal cysts [16]. If the ‘one-way valve’ flow was not corrected during surgery, the continuous unidirectional flow of joint fluid would have continued, thereby possibly leading to postoperative recurrence [17]. Injection of corticosteroids and local anaesthetic under ultrasound and fluoroscopic guidance to treat popliteal cysts has been reported to be safe and to reduce pain symptoms in the majority of patients [18], but injection of medicine could not dispose the intra-articular pathologies and eliminate the ‘one-way valve’ flow.

Arthroscopic treatment of popliteal cysts causes minimal trauma, allows for rapid patient recovery, and can eliminate the unidirectional flow mechanism [19–22]. In the treatment of popliteal cysts, intra-articular lesions were also disposed to reduce the recurrence rate of the cysts [23]. Enlargement of both transverse and vertical valve communication ports and correction of intra-articular lesions were effective in the treatment of symptomatic popliteal cysts [24]. Arthroscopy restored two-way communication between the cyst and the joint cavity via a minimally invasive approach, thereby eliminating the drivers of popliteal cysts [25]. Arthroscopic portals for treating popliteal cysts include double posteromedial portals and single posteromedial portal [25, 26]. A figure-of-four position and double posteromedial portals were used to achieve adequate enlargement of the valve communication port between the cyst and the joint cavity and complete excision of the cyst wall, which was effective and safe for treating popliteal cysts [25]. Few studies have compared the clinical effects of arthroscopic treatment of popliteal cysts between the DPP technique and the SPP technique.

In our study, arthroscopic treatment of popliteal cysts using DPP approaches under a figure-of-four position was able to achieve adequate enlargement of the valve communication port and complete excision of the cyst wall, and obtained satisfactory clinical results. The operation time depended largely on the surgeon’s experience and on the level of difficulty of the cyst wall resection. In this study, the DPP group required a longer operation time than the SPP group due to an additional auxiliary posteromedial needed to establish, but the recurrence rate of cysts in the DPP group was obviously lower than that in the SPP group. The SPP approach caused visual field blindness and limited operating space during the operation, and the entire cyst cavity could not be observed and was probably contacted [27]. The DPP approach could be used as the observation and operation approach mutually to widen the field of view, enlarge the valve communication port adequately and excise the cyst completely, thereby reducing the recurrence rate of cysts. In another retrospective study, the recurrence rate was significantly lower in the DPP group (0%) than in the SPP group (4%)[28], which was similar to our results.

Lee reported that through DPP method, cysts were completely disappeared in approximately 40% of patients, and the size was shrunken in 60% of patients [29]. We found that 68% of cyst disappearance and 32% of cyst shrinkage in the DPP group. Lee’s study and our study all illustrated that DPP method could effective resolve the problem of cysts and greatly reduce the recurrence rate of cysts. Besides, Lee also found that presence of degenerative cartilage lesion represented an associated risk factor for residual popliteal cysts [29]. Thereby, treatment of intra-articular lesions was of prime importance in the arthroscopic surgery.

After comparing knee functional scores of the DPP and SPP groups, there was no significant difference in the Lysholm score and R–L grades between the DPP group and the SPP group in this study. These results indicated that both DPP and SPP could achieve well knee functional scores, but the recurrence rate was significantly lower in the DPP group than in the SPP group. The reason of the discordance between cyst recurrence and clinical results was the existence of asymptomatic cysts. In other words, recurrent cysts do not mean poor clinical results. After arthroscopic surgery of popliteal cysts, the unidirectional flow mechanism was eliminated and two-way flow was established between articular cavity and cyst cavity. Most recurrent cysts were asymptomatic and had well clinical results.

The common complications of arthroscopic treatment of popliteal cysts include hematoma formation, fluid infiltration, poor wound healing, neurovascular injury, deep venous thrombosis and infection [10]. The severe postoperative complications after arthroscopic treatment were neurovascular injury and infection, but no neurovascular injury or infection occurred in this study. Kp et al. [30] reported 1 case of popliteal artery aneurysm after arthroscopic cystectomy of a popliteal cyst, which was an uncommon complication. The neurovascular bundle of the popliteal fossa located in the septum or slightly lateral portion of the popliteal fossa was the main neurovascular structure at risk during this operation, especially in patients whose cysts were located outside and around the blood vessels. It was suggested that the lateral wall of the cyst should not be planned during cyst wall resection to prevent damage to the popliteal artery [30]. The most common complications after arthroscopic treatment were hematoma and fluid infiltration [19]. In this study, two patients in the DPP group and one patient in the SPP group experienced fluid infiltration under the gastrocnemius muscle, which improved after pressure bandaging and cold compression. Before the end of the operation, the cyst site should be pressurized with multilevel dressing, and the tourniquet should be loosened after the dressing is completed, which could avoid postoperative hematoma and fluid infiltration in the joint space and tissue space to the maximum extent. In another study, 2 patients (3.8%) had complications after arthroscopic treatment of popliteal cysts, including one who developed deep vein thrombosis and one with hypoesthesia. To prevent occurrence of deep vein thrombosis, early quadriceps femoris exercises and ankle pump exercises are encouraged after surgery.

The influence of the cyst wall on treatment outcomes remains controversial. The cyst wall was considered to be histologically thickened hyaloid tissue that did not contain any synovial cell secreting synovial fluid [20]. Although some researchers considered that cyst wall resection not only lengthened the operation time, but also had the risk of damaging the blood vessels and nerves in the popliteal area [20, 31], the DPP approach was repeatedly reported to yield more satisfactory clinical results and decrease the rate of recurrence [25, 32–35]. In some circumstances, the septum in multilocular cysts might be related to postoperative recurrence of the cysts [26], because multilocular cysts have multiple cavities with a complete capsule for each cavity and simply expanding the cyst opening may lead to inadequate drainage. Therefore, we suggest that arthroscopic enlargement of the cyst opening with debridement of the cyst wall is an effective technique with the least recurrence.

Based on our experience, some experience points were suggested in arthroscopic internal drainage and cyst wall resection through the DPP approach under a figure-of-four position. First, the surgeon needed to accurately locate the posteromedial valvular opening and adequately expand it. Second, the cyst wall should be completely removed to reduce the recurrence rate, especially for separated cysts. Third, all operations should be performed under direct view, and the shaver should be stopped when the posterolateral adipose tissues are exposed to prevent vascular and nerve damage. Fourth, intraoperative attention should be given to avoid damaging the root of the medial meniscus. Finally, the operation time should be shortened as much as possible and the tension of the leg should be considered during the operation.

The limitations of this study are that the number of cases is small and the follow-up time is short. The next step of study requires a large sample prospective study and long-term follow-up.

## Conclusion

Arthroscopic treatment of popliteal cysts using DPP approaches was able to achieve adequate enlargement of the valve communication port and complete excision of the cyst wall, and obtained satisfactory clinical results. Although the DPP approach required a longer operation time than the SPP approach, the DPP approach resulted in a lower recurrence rate. Arthroscopic treatment of popliteal cysts using double posteromedial portals under a figure-of-four position was a safe and effective surgical method.

## Data Availability

Data sharing not applicable to this article as no datasets were generated or analyzed during the current study.

## Abbreviations

DPP: Double posteromedial portal
SPP: Single posteromedial portal
R–L: Rauschening–Lindgren
ACL: Anterior cruciate ligament
MRI: Magnetic resonance imaging
G: Gastrocnemius
P: Popliteal cyst
S: Semimembranosus
